# Rhenium uptake and distribution in phaeophyceae macroalgae, *Fucus vesiculosus*

**DOI:** 10.1098/rsos.160161

**Published:** 2016-05-11

**Authors:** B. Racionero-Gómez, A. D. Sproson, D. Selby, D. R. Gröcke, H. Redden, H. C. Greenwell

**Affiliations:** 1 Department of Earth Sciences, Durham University, Durham DH1 3LE, UK; 2 Department of Chemistry, Durham University, Durham DH1 3LE, UK

**Keywords:** rhenium, macroalgae, uptake, distribution, phytomining, bioremediation

## Abstract

Owing to Rhenium (Re) having no known biological role, it is not fully understood how Re is concentrated in oil kerogens. A commonly held assumption is that Re is incorporated into decomposing biomass under reducing conditions. However, living macroalgae also concentrate Re to several orders of magnitude greater than that of seawater. This study uses *Fucus vesiculosus* to assess Re uptake and its subsequent localization in the biomass. It is demonstrated that the Re abundance varies within the macroalgae and that Re is not located in one specific structure. In *F. vesiculosus*, the uptake and tolerance of Re was evaluated via tip cultures grown in seawater of different Re(VII) compound concentrations (0–7450 ng g^−1^). A positive correlation is shown between the concentration of Re-doped seawater and the abundance of Re accumulated in the tips. However, significant differences between Re(VII) compounds are observed. Although the specific cell structures where the Re is localized is not known, our findings suggest that Re is not held within chloroplasts or cytoplasmic proteins. In addition, metabolically inactivated *F. vesiculosus* does not accumulate Re, which indicates that Re uptake is via syn-life bioadsorption/bioaccumulation and that macroalgae may provide a source for Re phytomining and/or bioremediation.

## 1. Introduction

The behaviour of rhenium (Re) in seawater is defined by the low reactivity of the perrhenate ion (${\mathrm{ReO}}_{4}^{-}$; Re(VII)), which is the only significant Re species found in ocean waters [1]. The concentration of Re in the open ocean (0.0074–0.009 ng g^−1^; [2,3]) is a factor of three higher than average river water (approx. 0.005 pg g^−1^; [4]) and much lower compared with terrestrial environments (continental crust values of 0.2–2 ng g^−1^; organic-rich sedimentary rocks values 0.2–100 ng g^−1^; [5] and references therein) and sulfide minerals (low ng g^−1^ to hundreds of mg g^−1^; [6]).

Although the Re concentration in seawater is low in comparison with the terrestrial realm, and despite there being no known biological use of Re, marine macroalgae (i.e. seaweed), especially brown macroalgae, are known to concentrate Re up to several hundreds of ng g^−1^ [7–9], in addition to many metal cations and oxoanions through forming a variety of metal complexes with, for example, alginate, proteins, polysaccharides of the cell wall, fucans, etc. [10]. To date, positively charged metals associated with macroalgae have been extensively studied [11–14]; however, relatively little is known about the mechanisms by which macroalgae take up negatively charged metal oxoanions such as the perrhenate ion. Experiments have shown that Re is most likely stored within algal cells, rather than on the algal cell surface or within the intercellular matrix [9,15]. Specifically, it has been proposed that protonated amino groups could be involved, forming an ion pair with perrhenate [15,16]. Moreover, Kim *et al.* [17] showed that ${\mathrm{ReO}}_{4}^{-}$ interacted strongly with chitosan, a cationic polymer of glucosamine. Chitosan is only reported in nature in some fungi, crustacea and the termite queen's abdominal wall. However, Nishino *et al.* [18] isolated and characterized a novel polysaccharide containing an appreciable amount of glucosamine in *F. vesiculosus*, which suggests a further route to possible Re uptake.

Assuming that Re is being stored inside the macroalgae cells, a mechanism for Re uptake into the cells should be identifiable. Macroalgae could inadvertently take up ${\mathrm{ReO}}_{4}^{-}$ (ionic radius of 2.60 Å) by confusing it for phosphate (${\mathrm{PO}}_{4}^{3-}$; ionic radius of 2.38 Å). A similar mechanism is proposed for arsenate (${\mathrm{AsO}}_{4}^{3-}$) [19]. Sulfate (${\mathrm{SO}}_{4}^{2-}$), nitrate (${\mathrm{NO}}_{3}^{-}$) and chloride (Cl^−^) also have similar ionic radii to ${\mathrm{ReO}}_{4}^{-}$ (i.e. 2.58, 1.96 and 1.81 Å, respectively). Thus, these ions could be also competing with ${\mathrm{ReO}}_{4}^{-}$. For instance, Tagami & Uchida [20] showed that there is a positive correlation between K^+^ and technetium (Tc) accumulated in three plant species (*Cucumis sativus L., Raphanus sativus L.* and *Brassica chinensis L.*) and explained this as a result of ${\mathrm{TcO}}_{4}^{-}$ being taken up by mistaken identity for Cl^−^, as a counter ion for K^+^ uptake. As Re is a Tc analogue [9,17,21], ${\mathrm{ReO}}_{4}^{-}$ might be taken up in a similar manner. In addition, competitive incorporation between ${\mathrm{ReO}}_{4}^{-}$ and ${\mathrm{NO}}_{3}^{-}$ in sodalites has also been found [22]; however, as sodalite is a mineral, ${\mathrm{ReO}}_{4}^{-}$ incorporation cannot be compared with ${\mathrm{ReO}}_{4}^{-}$ concentration in biologically active organisms.

Importantly, understanding the uptake of Re will help to elucidate the uptake of Tc, which is produced in nuclear power stations. Moreover, a better knowledge on the uptake mechanism could open the possibility to use macroalgae as bioconcentrators of Re and Tc, thus bioremediation of Tc-contaminated waters and phytomining of Re could be achieved using *F. vesiculosus*, as well as potentially providing an alternative hypothesis for the high concentration of Re within oil-forming kerogens.

**Figure 1.**
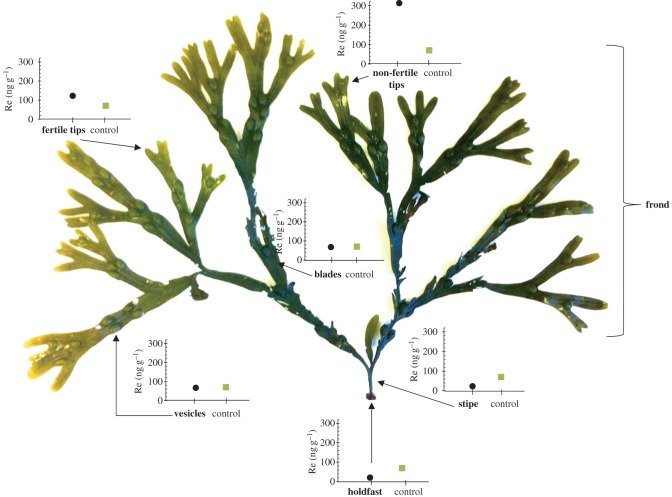
Average (two to five samples) concentration of rhenium (ng g^−1^) in the different structures of *F. vesiculosus*. Round marker symbolizes Re abundance in each particular structure and square marker symbolizes Re abundance of a mixture of all the structures (control). All the samples had a reproducibility of less than 5% RSD; in some cases, graph symbol size is greater than uncertainties. The concentrations shown are in dry mass, and although the concentration of each structure might change when wet mass, the differences of Re concentration are greater than the differences in water loss.

This study uses a brown macroalgae (Phaeophyceae) to establish: (i) where Re is stored; (ii) the limit of Re uptake; and (iii) the uptake mechanism of Re (i.e. active concentration in which the transport requires energy to oppose the concentration gradient, or passive concentration, with transport requiring no energy and entirely correlated with the concentration). The Re abundance data for the different structures of *F. vesiculosus*: holdfast, stipe, fertile tips, non-fertile tips, vesicles and blades (figure 1), and isolated cytoplasmic proteins and chloroplasts are investigated. The uptake limit of Re in macroalgae is determined via cultures of *F. vesiculosus* under different ${\mathrm{ReO}}_{4}^{-}$ concentrations and using different ${\mathrm{ReO}}_{4}^{-}$ chemical compounds (i.e. HReO_4_ (Re metal dissolved in HNO_3_), KReO_4_, NaReO_4_ and NH_4_ReO_4_). Cultured versus dead macroalgae were used to provide insights into the uptake mechanism of ${\mathrm{ReO}}_{4}^{-}$ in macroalgae.

## 2. Material and methods

### 2.1. Macroalgae used in the study: *Fucus vesiculosus*

The available Re data for brown macroalgae (Phaeophyceae) indicate it has the highest Re accumulation of all macroalgae, with *Fucus vesiculosus* possessing the highest Re concentrations measured to date for a macroalgae [7]. *F. vesiculosus* is a common macroalgae found along sheltered shores of the North Sea, Baltic Sea, Atlantic Ocean and Pacific Ocean. *F. vesiculosus* is a tethered macroalgae with air bladders that are produced annually allowing the individual fronds to float. The growth rate ranges between 0.05 and 0.14 cm d^−1^ [23,24] and they have a lifespan in the order of 3–5 years [25]. The species is annually episodic, gonochoristic and highly fecund (i.e. prolific) [25]. Gametes are released into the seawater, and the eggs are fertilized externally to form a zygote that starts to develop as soon as it settles into a substrate [26]. The gametes are released from receptacles, which are found in the fertile tips of the macroalgae. However, *F. vesiculosus* also has non-fertile tips without these structures. Non-fertile tips are composed by a parenchymatous thallus (i.e. tissue-like structure) [25–27]. The structures of *F. vesiculosus* are shown in figure 1.

### 2.2. Macroalgae collection sites

Five specimens of *F. vesiculosus* were collected from Staithes, North Yorkshire, UK (54°33′ N 00°47′ W) in May 2014. These samples were used to determine the Re abundance of specific structures of the macroalgae. An additional six samples were collected each month at Boulmer Beach, Northumberland, UK (55°25′ N 1°34′ W) in May, June, October and November in 2014, and January to June in 2015, for fertile and non-fertile tip separation, all the culture experiments, chloroplast isolation and protein purification.

### 2.3. Rhenium abundance and distribution in macroalgae structures

Prior to analysis, all specimens were kept individually in plastic sample bags for transport, and stored in a freezer (−10°C) for 48 h. Each specimen was washed and soaked in deionized (Milli-Q™) water to remove any attached sediment and salt. To establish the abundance and distribution of Re in the macroalgae, the sample was divided into different structural components; fertile tips, non-fertile tips, vesicles, stipe, holdfast, blades (figure 1). In addition, all the algae components were mixed to assess an average Re abundance. Each structure was dried in an oven at 60°C for 12 h.

### 2.4. Rhenium uptake of macroalgae

**Figure 2.**
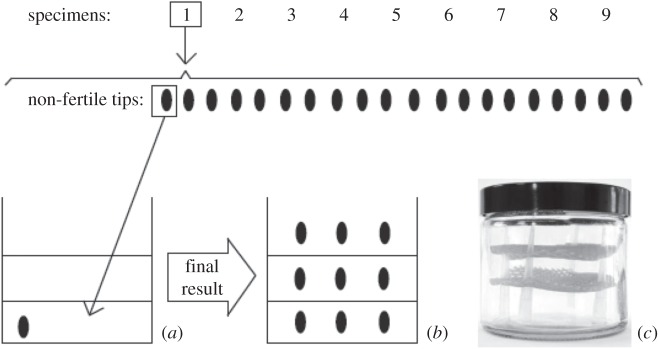
Culture representation of non-reproductive *F. vesiculosus* thallus tips. Twenty-one tips of each *F. vesiculosus* specimen were cut and a tip from each specimen was displaced into one of the 21 jars (*a*). Two meshes were put inside each jar ending up with three levels that store three non-fertile tips each (*b*). (*c*) Real culture jar picture.

To investigate the uptake of Re by macroalgae, non-reproductive apical thallus tips of nine *F. vesiculosus* specimens (length = greater than 1.5 cm; wet weight (WW) = 0.12–0.15 g), without visible microalgae (i.e. epiphytes), from Boulmer Beach were cultured in seawater (modified after Gustow *et al*. [28]) with a known concentration of Re. In brief, the culture experiments were performed using a 250 ml glass jar containing two mesh shelves. Three tips were placed in the bottom of the jar and three tips to each mesh, having in total nine tips, with each set of tips taken from a different specimen (figure 2). All jars were filled with sterile filtered (0.7 µm) seawater from Boulmer Beach. A huge diversity of macroalgae grow naturally at Boulmer Beach, thus water obtained at Boulmer water is expected to be nutrient replete as it permits the growth of a wide variety of species. Each set of three jar replicates were doped using a known volume of ${\mathrm{ReO}}_{4}^{-}$ from different Re compounds: an already prepared solution of Re metal with nitric acid (HReO_4_; i.e. 83787 Sigma Aldrich) or commercially obtained Re(VII) salts (KReO_4_, NH_4_ReO_4_ and NaReO_4_).

HNO_3_ dissolves Re metal forming HReO_4_ [29]. For the cultures using HReO_4_, Boulmer seawater ${\mathrm{ReO}}_{4}^{-}$ concentration was analysed. The Re abundance in the seawater was determined by isotope dilution inductively coupled plasma mass spectrometry (ICP–MS) (details below). The seawater possesses a Re abundance of approximately 0.007 ng g^−1^ (6.95 ± 0.19 pg g^−1^) coinciding with the concentrations reported by Anbar *et al.* [2]. The seawater culture experiments were conducted in Re concentrations equal to that of seawater, and 10×, 50×, 100×, 500×, 1000×, 2667×, 10 000×, 133 333× and 266 667× that of the concentration of seawater (i.e. 0.007, 0.075, 0.373, 0.745, 3.725, 7.450, 20, 75, 1000 and 2000 ng g^−1^, respectively). In addition, three jars were filled with artificial seawater that was not doped with Re, and one jar was doped with a concentration a million times that of the Re seawater concentration in order to reach an extreme concentration of 7450 ng g^−1^.

For the cultures using Re(VII) (perrhenate) salts, the same approach was used, where the doped Re concentrations of seawater in the cultures were 10×, 50×, 100× and 1000× that of seawater (i.e. 0.075, 0.373, 0.745 and 7.45 ng g^−1^, respectively).

To reduce evaporation, while allowing gaseous exchange with the atmosphere, all the jars were loosely covered with lids. No additional nutrients were added into the seawater or artificial seawater. The algae tips inside the bottles were transferred into an incubator with a set light/dark rhythm of 16 : 8, light intensity of 125 µmol photonsm^−2^ s^−2^ and a temperature of 11°C. The WW of the algal tips, per jar, was measured every 2–3 days during 25 days of the culturing period for all cultures except the cultures of June 2015, which only lasted 15 days. At the same time, the media were changed (between four and seven times for all cultures) to avoid accumulation of metabolites and replenish nutrients. The salinity (approx. 35 ppt) of the Re-doped seawater did not appreciably change from that of natural seawater collected from Boulmer and remained constant throughout the culture experiments. The pH (approx. 9.0), however, changed from that of the natural seawater collected from Boulmer (approx. 8.2) owing to the metabolic activity of the macroalgae (photosynthesis) and remained constant throughout the culture experiments.

Two additional sets of culture experiments were conducted to establish if ${\mathrm{ReO}}_{4}^{-}$ is taken up by syn-life bioabsorption/bioaccumulation or passive processes. Understanding syn-life bioaccumulation and bioabsorption as the biological sequestration of substances or chemicals through any route at a higher concentration than that at which it occurs in the surrounding environment/medium when macroalgae is metabolically active (i.e. alive) [30]. Therefore, in order to assess bioaccumulation, non-reproductive thallus tips were killed through either boiling, drying or freezing. Specifically, non-reproductive thallus tips (*n* = 81) from Boulmer Beach were heated for 2 h at 100°C, and a further 21 tips were heated at 100°C for only 5 min. Additionally, 21 non-reproductive thallus tips were air dried for 72 h, and another 21 tips were frozen with liquid nitrogen. In total, 18 jars were filled with sterile (i.e. autoclaved at 121°C for 30 min) and filtered (0.7 µm) seawater from Boulmer Beach. The jars containing boiled tips were divided into three subgroups composed of three replicates of each with the following treatments: seawater and seawater doped with 7.45 ng g^−1^ of HReO_4_. The other set of three replicates containing dried, boiled (5 min) or frozen non-reproductive thallus tips, respectively, were only treated with seawater spiked with 7.45 ng g^−1^ HReO_4_.

In order to reconfirm the uptake mechanism, four tips were placed in the bottom of the jar and four tips to each mesh, having in total 12 tips of different specimens in each jar. All jars were filled with sterile filtered (0.7 µm) seawater from Boulmer Beach and doped with 7.45 ng g^−1^ NaReO_4_. After 3 days, the media solution was changed and set to 0.075 ng g^−1^ of NaReO_4_ and, finally, after another 3 days, the media solution was changed and not doped. Prior to each change of the media four sample tips were taken for Re analysis.

### 2.5. Chloroplast isolation

A procedure modified from Popovic *et al.* [31] was used for the isolation of chloroplasts. Approximately 10 g of non-reproductive thallus tips were cut into 2 mm^2^ pieces using scissors. These were washed by stirring with 2 l of filtered seawater with 75 ml of grinding medium added. The grinding medium consisted of 1 M sorbitol, 2 mM MnCl_2_, 1 mM MgCl_2_, 0.5 mM K_2_HPO_4_, 5 mM EDTA, 2 mM NaNO_3_, 2 mM ascorbate, 2 mM cysteine, 0.2% (w/v) BSA and 50 mM of MES buffer (pH 6.1). All the subsequent steps were undertaken in ice water. The washed tissue was divided into two portions, each ground with a mortar and pestle, increasing gradually the volume to 50 ml. Then, each portion was diluted into 100 ml of medium and passed through a stainless steel strainer and four layers of cheese cloth. Chloroplasts were isolated by centrifugation for 7 min at 5500*g*. The pellet was resuspended with 10 ml of a reaction medium containing 1 M sorbitol, 1 mM MnCl_2_, 1 mM MgCl_2_, 2 mM EDTA, 0.5 mM K_2_HPO_4_ and 50 mM HEPES (pH 8.1). Another centrifugation at 5500*g* for 7 min was performed, and chloroplasts were re-suspended with 2 ml of HEPES buffer. To test the isolation, the absorbance spectrum of the last solution obtained was observed under a light microscope. The extracted chloroplasts were preserved using HEPES (as it does not contain Re) and stored in a fridge for 3 days. In order to remove HEPES from the chloroplasts, the HEPES–chloroplast mixture was centrifuged. The chloroplast pellet was white–brown, and the HEPES solution was green–brown. The observation showed that the pigments had released and were free in the solution.

### 2.6. Cytoplasmic proteins isolation

A procedure modified from Boer *et al.* [32] was employed for the isolation of cytoplasmic proteins. Approximately 2 g of freshly ground non-reproductive thallus tips were used for protein extraction. The tips were mixed with 9 ml of 10 mM HEPES (pH 7.8) buffer, vortexed and centrifuged twice at 1000*g* for 1 min. The homogenate was sonicated for 1 min, 10 times and centrifuged at 4500*g* for 5 min. The supernatant was centrifuged at 14 000*g* for 10 min. A 60 mM saturated CaCl_2_ solution was used to re-suspend the pellet, which was agitated and then centrifuged at 14 000*g* for 5 min. The supernatant was then separated via gel filtration (i.e. size exclusion column chromatography). A PD-10 desalting column containing *Sephadex G-25 medium* as matrix was used to separate molecules from the supernatant by their molecular size. Larger molecules than the *Sephadex* matrix pores are eluted first and smaller molecules than the matrix pores are eluted later, depending on the molecular size, the molecules will penetrate the matrix pores to varying extent. The separation was carried out following the gravity protocol detailed in PD-10 Desalting Columns Instructions [33] using the same buffer described above. Of 1 ml elution fractions obtained were analysed by ICP–MS after being diluted 10 times with 0.8 N HNO_3_. Protein content of the fractions was analysed based on the absorbance shift of the dye Coomassie brilliant blue G-250.

### 2.7. Re abundance determinations and data treatment

Rhenium abundance determinations for all samples were obtained at the Durham Geochemistry Centre in the Laboratory for Sulfide and Source Rock Geochronology and Geochemistry. Each sample of *F. vesiculosus* was oven-dried at 60°C for 24 h and ground into a powder with an agate mortar and pestle. Approximately 100 mg of the sample powder was spiked. Abundances were obtained by both direct calibration and isotope dilution methodologies (tables 1–5). For the latter, samples were doped with a known amount of ^185^Re tracer solution (isotope dilution methodology). The sample and, if used, the tracer solution were digested in a mix of 3 ml of 12 N HCl and 6 ml of 16 N HNO_3_ at 120°C overnight in a PFA Savillex 22 ml vial. The dissolved sample solution was evaporated to dryness at 80°C. The rhenium abundance of seawater from Boulmer Beach was determined by isotope dilution ICP–MS. Approximately 30 ml of seawater was doped with a known amount of ^185^Re tracer solution and evaporated. The rhenium fraction was further purified using standard anion chromatography methodology. Rhenium for all macroalgae samples was isolated from the dried sample using 5 ml 5 N NaOH 5 ml acetone solvent extraction procedure [8,34]. The Re-bearing acetone was evaporated to dryness at 60°C. For ICP–MS, the dried Re fraction was dissolved in 1.2 ml of 0.8 N HNO_3_. For thermal ionization mass spectrometry in negative ion mode analysis, the purified Re fraction was loaded onto a Ni wire filament, with the Re isotope compositions determined using Faraday cup measurements on a Thermo Scientific TRITON mass spectrometer. Total procedural blanks are 1 ± 0.1 pg (*n* = 6). For samples analysed by isotope dilution to determine absolute Re abundance, all sources of uncertainty (e.g. standard measurement, isotope measurement, calibration of the tracer solution, fractionation correction and blank values) are propagated to yield a final uncertainty. For direct calibration, prior to each analysis, instrument performance checks confirm satisfactory performance of the ICP–MS. Five freshly prepared standards were made each time and formed calibration lines with an *R*-value more than 0.999 and less than 2% RSD uncertainty. Moreover, all the samples had a reproducibility of less than 5% RSD.

**Table 1.** Re abundance for *F. vesiculosus* structures analysed with Thermo Scientific X-series ICP–MS isotope dilution methodology.

sample	Re (ng g^−1^)	2*σ* (±)
*macroalgae 1*		
control	69.8	0.1
tips 1	163.4	0.1
leaves	28.4	0.1
stipe	23.0	0.2
holdfast	21.0	0.2
blades	67.3	0.1
veins	33.8	0.1
blades without veins	65.8	0.1
*macroalgae 2*		
fertile tips	117.4	<0.1
non-fertile tips	383.2	<0.1
tips	76. 0	0.1
control	51.0	0.1
*macroalgae 3*		
fertile tips	145.0	<0.1
non-fertile tips	363.2	<0.1
tips	144.1	<0.1
control	103.4	0.1
*macroalgae 4*		
fertile tips	106.4	0.1
non-fertile tips	273.5	<0.1
tips	158.5	0.1
control	61.0	0.1
*macroalgae 5*		
fertile tips	120.7	0.1
non-fertile tips	229.1	<0.1
tips	147.2	0.1
control	84.3	0.1
*macroalgae 6*		
non-fertile tips	382.5	<0.1
fertile tips	129.5	0.1
tips	105.1	0.1
*macroalgae 7*		
control^a^	64.0	0.7
tips^a^	138.0	0.7
blades^a^	56.8	0.3
stipe^a^	22.5	0.2
holdfast^a^	21.6	0.2
blades2^a^	58.9	0.4

a Samples analysed with Thermo Scientific Triton Mass Spectrometer.

**Table 2.** Re concentrations of the media used for Re uptake experiments for boiled (2 h and 5 min), dried, and frozen with liquid nitrogen *F. vesiculosus* tips. Re abundances determined with Thermo Scientific X-series ICP–MS isotope calibration methodology.

non-reproductive thallus tips treatment	Re (ng g^−1^) doped in seawater media previously	Re (ng g^−1^) in seawater media afterwards	2*σ* (±)
*boiled*			
2 h	7.5	7.1	0.0
5 min	7.5	7.1	0.1
dried 72 h	7.5	2.6	0.0
frozen with N_2_ liquid	7.5	6.6	0.0
non-treated macroalgae (control)	7.5	0.3	0.0

**Table 3.** Re concentrations of the boiled (2 h and 5 min), dried, and frozen with liquid nitrogen *F. vesiculosus* tips following Re uptake experiments. Re abundances determined with Thermo Scientific X-series ICP–MS isotope calibration methodology.

non-reproductive thallus tips treatment	Re (ng g^−1^) doped in seawater media	Re (ng g^−1^) uptaken by *F. vesiculosus*	2*σ* (±)
*boiled*			
2 h	7.5	36.2	0.1
2 h	0.0075	1.1	1.0
2 h	0.0	0.5	1.0
5 min	7.5	20.9	<0.1
dried 72 h	7.5	24.1	<0.1
frozen with N_2_ liquid	7.5	20.0	<0.1

**Table 4.** Re concentration of macroalgae tips cultured under the different concentrations of HReO_4_ in the media. Re abundances determined with Thermo Scientific X-series ICP–MS with isotope calibration methodology.

replicate number	HReO_4_ (ng g^−1^) seawater	Re (ng g^−1^) uptake by *F. vesiculosus*	2*σ* (±)	replicates average	SD (±)
1	0.0075	187.0	0.4	168.2	9.5
2	0.0075	149.4	0.2		
1	0.07	549.6	0.2	415.4	50.6
2	0.07	391.0	0.1		
3	0.07	305.7	1.0		
1	0.4	995.2	16.0	1275.6	135.2
2	0.4	1190.0	1.3		
3	0.4	1641.7	52.0		
1	0.8	1668.1	0.3	1769.6	84.4
2	0.8	2007.3	3.0		
3	0.8	1633.3	2.4		
1	3.7	8575.0	18.1	9218.6	455.1
2	3.7	10 505.9	2.9		
3	3.7	8575.0	12.8		
1	7.5	15 961.8	37.9	16 208.7	90.1
2	7.5	16 387.0	5.0		
3	7.5	16 277.3	50.2		
1	20.0	48 738.7	69.0	48 007.2	2009.2
2	20.0	52 521.9	74.0		
3	20.0	42 760.9	68.0		
1	75.0	51 477.0	72.0	63 283.4	5718.7
2	75.0	59 611.8	16.5		
3	75.0	78 761.5	99.0		
1	1000.0	53 009.5	45.0	55 588.2	2188.9
2	1000.0	61 752.1	85.5		
3	1000.0	52 003.1	99.5		
1	2000.0	23 488.8	4.0	22 472.5	512.0
2	2000.0	21 070.8	26.5		
3	2000.0	22 857.8	16.0		
1	7450.0	33 061.0	50.0	33 061

**Table 5.** Re concentration of macroalgae tips cultured under the different concentrations of Re(VII) salts and HReO_4_ in the media. Re abundances determined with Thermo Scientific X-series ICP–MS with isotope calibration methodology.

replicate number	NaReO_4_ (ng g^−1^) seawater (March)	Re (ng g^−1^) uptake by *F. vesiculosus*	2*σ* (±)	replicates average	SD (±)
2	0.074	206.3	0.2	219.6	6.6
3	0.074	232.9	0.5		
2	0.373	624.5	0.8	629.5	2.5
3	0.373	634.5	1.0		
2	0.745	986.7	2.3	1033.6	23.4
3	0.745	1080.4	2.1		
2	7.450	8421.4	6.3	8064.2	178.6
3	7.450	7706.9	11.5		

replicate number	NaReO_4_ (ng g^−1^) seawater (May)	Re (ng g^−1^) uptake by *F. vesiculosus*	2*σ* (±)	replicates average	SD (±)
2	0.0074	95.3	<0.1	86.1	4.6
3	0.0074	76.9	<0.1		
2	0.074	175.0	<0.1	132.9	21.0
3	0.074	90.9	<0.1		
2	0.373	214.3	0.1	200.3	7.0
3	0.373	186.4	0.1		
2	0.745	227.9	0.3	225.7	1.1
3	0.745	223.5	0.2		
2	7.450	1268.0	1.1	1203.9	32.0
3	7.450	1139.9	1.7		

replicate number	NH_4_ReO_4_ (ng g^−1^) seawater (May)	Re (ng g^−1^) uptake by *F. vesiculosus*	2*σ* (±)	replicates average	SD (±)
2	0.074	230.6	<0.1	226.1	2.2
3	0.074	221.6	<0.1		
2	0.373	128.6	<0.1	129.4	9.4
3	0.373	130.1	<0.1		
2	0.745	283.6	<0.1	268.9	7.3
3	0.745	254.3	0.1		
2	7.450	1244.6	0.3	1208.1	18.2
3	7.450	1171.6	2.1		

replicate number	KReO_4_ (ng g^−1^) seawater (May)	Re (ng g^−1^) uptake by *F. vesiculosus*	2*σ* (±)	replicates average	SD (±)
2	0.074	88.0	0.1	91.9	7.0
3	0.074	95.9	0.1		
2	0.373	143.6	<0.1	138.4	2.6
3	0.373	133.2	0.1		
2	0.745	166.5	<0.1	176.1	4.8
3	0.745	185.8	0.3		
2	7.450	1260.3	0.5	1251.1	4.4
3	7.450	1242.2	0.6		

replicate number	NH_4_ReO_4_ (ppb) seawater (May)	Re (ppb) uptake by *F. vesiculosus*	2*σ* (±)	replicates average	SD (±)
2	0.074	81.0	0.2	82.3	0.7
1	0.074	83.7	<0.1		
2	0.745	125.4	0.2	129.2	1.9
1	0.745	133.0	<0.1		
2	7.450	689.2	3.3	732.8	21.8
1	7.450	776.4	0.2		
2	0.074	51.9	0.1	58.3	3.2
1	0.074	64.6	<0.1		
2	0.745	233.8	0.6	272.4	2.2
1	0.745	242.6	1.0		
2	7.450	587.0	0.4	564.9	10.7
1	7.450	544.4	<0.1		

replicate number	HReO_4_ (ng g^−1^) seawater (June)	Re (ng g^−1^) uptake by *F. vesiculosus*	2*σ* (±)	replicates average	SD (±)
2	0.074	125.6	<0.1	128.6	1.5
1	0.074	131.8	<0.1		
2	0.745	733.79	0.2	722.5	5.6
1	0.745	711.3	41.0		
2	7.450	5924.3	33.5	6741.4	408.6
1	7.450	7558.6	56.5		

Statistical analysis, *t*-test and Tukey's HSD tests, using a significance level of 0.05, were performed using R Studio software. For testing the statistical hypothesis, *p*-values are used. The *p*-value is defined as the probability of obtaining a result more extreme or equal to what was actually observed, thus, if *p*-value is smaller or equal to the significance level, it suggests that the observed data are consistent with the hypotheses.

## 3. Results

### 3.1. Location of Re within *Fucus vesiculosus* structures

All analysed structures of *F. vesiculosus* are naturally enriched in Re by approximately 1000 times that found in seawater (figure 1). The contents of Re range from 23 to 313 ng g^−1^ (figure 1). Significant differences were observed (*p*-value: 0.02) between the five samples of macroalgae tips (approx. 126 ng g^−1^) and the sample representing a mix of the plant components (approx. 74 ng g^−1^). Further, significant differences were also observed (*p*-value: 0.003) between fertile (approx. 123 ng g^−1^) and non-fertile tips (approx. 313 ng g^−1^; figure 1).

### 3.2. Uptake of Re by *Fucus vesiculosus* culture tips

**Figure 3.**
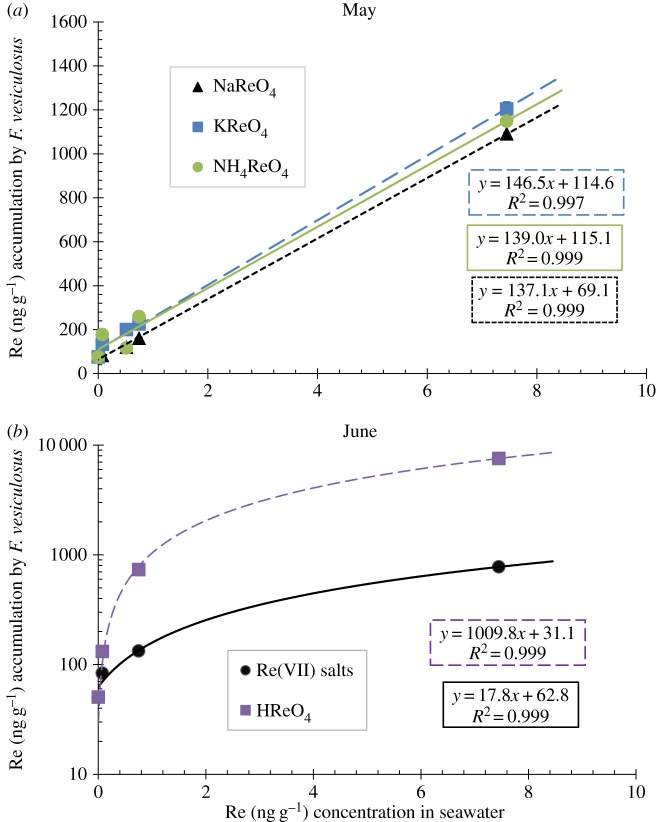
(*a*) Rhenium (ng g^−1^) accumulation in *F. vesiculosus* under different Re(VII) salts concentrations. Cultures made with NH_4_ReO_4_ represented with a round marker, KReO_4_ with a square marker and NaReO_4_ with a triangle marker. (*b*) Rhenium (ng g^−1^) accumulation in *F. vesiculosus* under different Re(VII) salts (round marker) and HReO_4_ (square marker) plotted in logarithmic scale. All the samples had a reproducibility of less than 5% RSD; in some cases, graph symbol size is greater than uncertainties.

**Figure 4.**
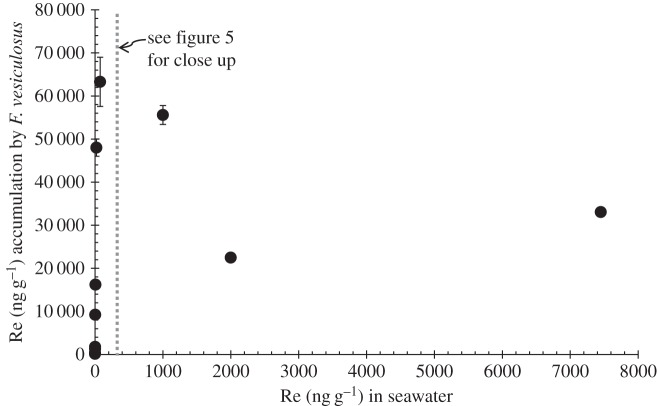
Rhenium (ng g^−1^) accumulation in *F. vesiculosus* under different HReO_4_-doped seawater concentrations. It follows a logarithmic trend line. All the samples had a reproducibility of <5% RSD; in some cases, graph symbol size is greater than uncertainties.

**Figure 5.**
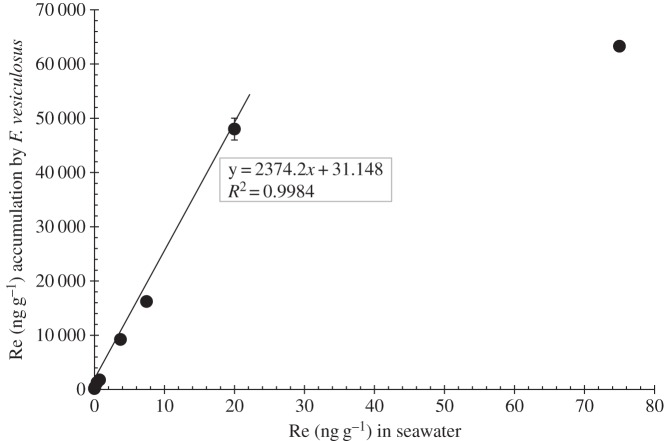
Rhenium (ng g^−1^) accumulation in *F. vesiculosus* under different HReO_4_-doped seawater concentrations. All the samples had a reproducibility of <5% RSD; in some cases, graph symbol size is greater than uncertainties.

The natural Re abundance of the seawater collected from Boulmer Beach and used for the culture experiments is 6.95 ± 0.19 pg g^−1^ (approx. 0.007 ng g^−1^), which is in agreement with previous studies of coastal waters [2]. The results shown in figures 3–5 indicate that in 25 days the Re content of the macroalgae increased proportionally to the amount of Re species doped in the seawater. However, variation in the uptake capacity by *F. vesiculosus* of the different ${\mathrm{ReO}}_{4}^{-}$ compounds doped in seawater is observed. Moreover, a significant variation (*p*-value less than 0.05) in uptake capacity between months of collection (i.e. February, March, May and June cultures with Re(VII) salts) was observed only after 0.37 ng g^−1^ of doped Re(VII) in the media. March cultures accumulated approximately 7000 ng g^−1^ more Re than February, May and June culture tips (table 6). Moreover, cultures doped with HReO_4_ and Re(VII) salts also show different amounts of accumulation. The accumulation of Re in *F. vesiculosus* grown with all Re(VII) salts is significantly lower (*p*-value less than 0.05) than the accumulation obtained with cultures made with HReO_4_, also only after 0.37 ng g^−1^ of doped Re to the media (figure 3). It is observed that cultures in Re-doped solution made from HReO_4_ take up 50% of the amount of Re in seawater, in contrast to only 0.03–15% for solution doped with Re from Re(VII) salts (table 6). Because of this, cultures with high concentrations of ReO_4_ in the media were made only with HReO_4_. A linear correlation is observed between the amount of Re doped in the cultures and the accumulation of Re in the alive cultured macroalgae until an accumulation of 63 284 ng g^−1^ of Re was reached, after which Re uptake ceased as the macroalgae died (figure 4). We also observed there is a limit on the uptake of Re in the cultured macroalgae between 75 and 1000 ng g^−1^ of HReO_4_ in the seawater media. Furthermore, visually, the macroalgae tips grown in high concentrations (2000 and 7450 ng g^−1^) did not seem as metabolically active as those in lower concentrations. In total, macroalgae tips extracted up to approximately 60 000 ng g^−1^ of Re in 25 days (figures 4 and 5).

**Table 6.** Seasonal uptake percentage variation of Re(VII) salts (i.e. NH_4_ReO_4_, KReO_4_ and NaReO_4_) cultures done in 2015 versus uptake rate of HReO_4_ cultures performed in June 2014 and 2015.

	Re(VII) salts				HReO_4_	
	February 2015	March 2015	May 2015	June 2015	June 2014	June 2015
number of media changes	5	5	7	4	5	4
total ReO_4_ (ng) in seawater [doped ng × number of media changes]	12 500	12 500	17 500	10 000	9300	7440
possible Re (ng g^−1^) accumulation by *F. v*.^a^	∼25 000	∼25 000	∼35 000	∼20 000	∼18 600	∼14 880
real Re (ng g^−1^) accumulation by *F.v.*	∼1700	∼8000	∼1200	∼800	∼9300	∼7400
% uptake [real/possible accumulation]	6.80	32.00	3.40	4.00	50.00	49.70

a Total Re in seawater/average dry weight of macroalgae tips (0.5 g).

**Figure 6.**
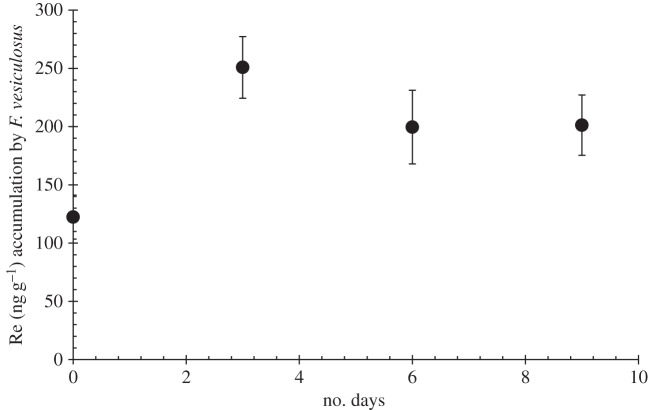
Re (ng g^−1^) accumulation in *F. vesiculosus* under changing concentrations of Re(VII) salts in the media. From day 1 to 3, Re concentration of 7.45 ng g^−1^; from day 3 to 6, 0.075 ng g^−1^; and from day 6 to 9, 0.0075 ng g^−1^. Day 0 measure is the background concentration of Re found in the seaweed cultured. All the samples had a reproducibility of <5% RSD.

*Fucus vesiculosus* non-fertile tips under 7.45 ng g^−1^ of NaReO_4_ in the media, after 3 days were capable of accumulating approximately 150 ng g^−1^ more than the background Re concentration in them (figure 6). These tips were then transferred to subsequent lower concentrations of NaReO_4_ (0.075 and 0.007 ng g^−1^) and exhibited accumulations of approximately 100 ng g^−1^ more than the background concentration of Re. Therefore, a release of 50 ng g^−1^ was found after transference (figure 6).

**Figure 7.**
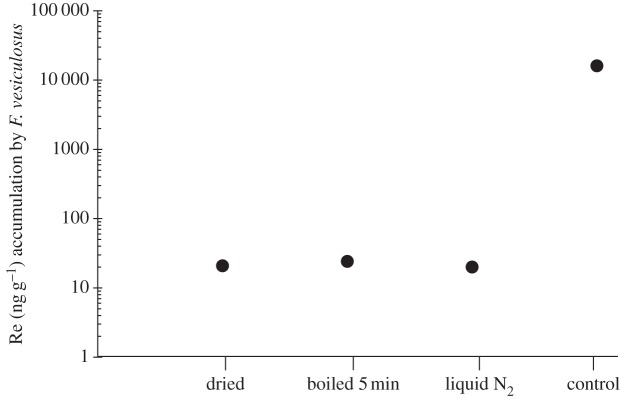
Accumulation of ${\mathrm{ReO}}_{4}^{-}$ in *F. vesiculosus* under different treatments (previously heated at 100°C for 5 min, liquid nitrogen frozen, and 30°C dried) and 7.45 ng g^−1^ HReO_4_ media concentration. All the samples had a reproducibility of <5% RSD.

In comparison with living organism samples, *F. vesiculosus* non-fertile thallus tips metabolically deactivated by boiling, freezing with liquid nitrogen or drying showed appreciably little to no accumulation of Re (between 36 and 19 ng g^−1^) compared with the concentration reached in fresh tips (i.e. alive; approx. 16 000 ng g^−1^) with the same HReO_4_ concentrations in the media of 7.45 ng g^−1^ (figure 7). Also, the majority of the Re content in the macroalgae was released in the media within the first 2–3 days of the experiment, and the media turned brown.

### 3.3. Chloroplast isolation

Chloroplasts were isolated from *F. vesiculosus* non-fertile tips. The non-fertile tips as a whole contain between 100 and 200 ng g^−1^ of Re. Chloroplasts are found throughout the whole macroalgae organism, although they exist in greater abundance in the non-fertile tips. Both the HEPES solution and the chloroplast pellet were analysed. In the chloroplast extract, 1 ng g^−1^ of Re was detected, and 3 ng g^−1^ of Re was detected in the HEPES solution in which the chloroplasts were stored (table 7). Regardless of the difficulty in isolating the chloroplast, less than 1% of the Re is present in the chloroplast relative to the host structure (non-fertile tips) which possesses approximately 150 ng g^−1^.

**Table 7.** Concentration of Re (ng g^−1^) in chloroplasts and in HEPES solution where chloroplasts were stored.

sample	Re concentration (ng g^−1^)
chloroplast pellet	∼1
HEPES solution	∼3

### 3.4. Cytoplasmic proteins purification

**Figure 8.**
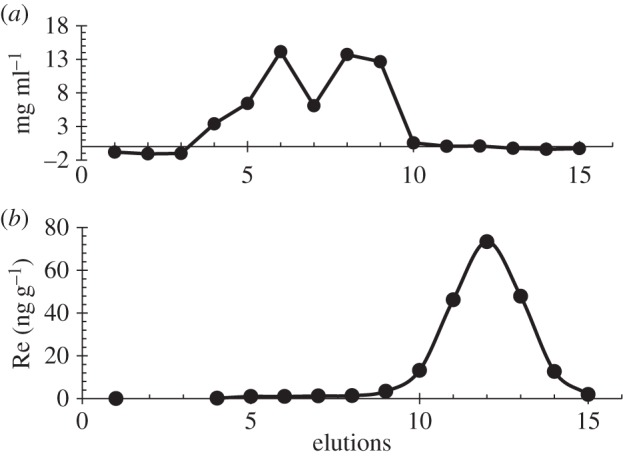
(*a*) Concentration of proteins (µg ml) in each elution (i.e. fraction eluted, corresponding to 1 ml). There are two protein peaks in elutions 6 and 8–9. (*b*) Concentration of rhenium (ng g^−1^) in each elution. The peak is in elution 12.

Cytoplasmic proteins (approx. 48 µg) were purified from 2 g of wet (i.e. 0.6 g dry) *F. vesiculosus* non-fertile tips. Proteins possess sizes in excess of 5 kDa, and were only found in fractions 4–6 eluting (1 ml fractions were collected with a G25 column). No Re was observed in the elutions containing the proteins (figure 8). However, a total amount of approximately 200 ng of Re was removed from the chromatography from elutions 10–14 with other unknown particles smaller than 5 kDa. Given the total volume of macroalgae used for the isolation of the protein (i.e. 0.6 g of dry weight), this equates to a concentration of approximately 300 ng g^−1^ Re, as it is between the range of Re expected to be in the non-fertile tips, it can be stated that all Re from the tips structures was eluted.

## 4. Discussion

### 4.1. Localization of Re within *Fucus vesiculosus* structures

The apical growth in the Phaeophyceae family is thought to occur by division of cells in cylindrical directions, with daughter cells generating a parenchymatous tissue construction [26]. Parenchyma tissue cells are capable of cell division if stimulated and can differentiate into specialized cells for photosynthesis, reproduction, growth and nutrient uptake. In Phaeophyceae, it is possible to distinguish five types of cells: epidermal cells, primary cortical cells, secondary cortical cells, medullary cells and hyphae [35]. The non-fertile tips are the apical meristems of *F. vesiculosus*, therefore, they are composed of cells that can divide and differentiate, including photosynthetic cells. Although there is variability between the different macroalgae specimens collected, the relative levels of Re vary significantly within the macroalgae structures. There are significant differences (*p*-value less than 0.05) between the amount of Re stored in the tips (approx. 126 ng g^−1^) versus Re stored in the remainder of the macroalgae (approx. 74 ng g^−1^; figure 1). Furthermore, significant concentration of Re is found in the non-fertile tips, which suggests a link between Re and the meristematic and photosynthetic specialized cells. More specifically, an average concentration of 313 ng g^−1^ of Re was found in the non-fertile tips, 122 ng g^−1^ Re in the fertile tips, 67 ng g^−1^ Re in the blades, 66 ng g^−1^ Re in the vesicles, 23 ng g^−1^ Re in the stipe and 21 ng g^−1^ Re in the holdfast. This suggests that Re is most likely stored in the photosynthetic structures, and it is not involved in the reproductive structures (receptacles). In herbaceous plants, the distribution of Re is also higher in photosynthetic structures, with 86% of the plant Re reported to be at the leaves [36]. Bozhkov & Borisova [37] stated that, in plants, Re is accumulated in chlorophylls forming Mg(ReO_4_)_2_. However, no Re was found in the chloroplasts of *F. vesiculosus*, thus our study suggests that Re is not strongly bound by/to chlorophylls. The concentrations of Re in the chloroplast extraction and the HEPES solution where the chloroplasts were stored are 1 and 3 ng g^−1^ of Re, respectively (table 7). These concentrations are very low, much lower than the concentrations expected given the observed concentration on the tip structures (approx. 100 ng g^−1^).

It should be emphasized that the data in table 1 show that there is Re in all parts of *F. vesiculosus*, i.e. Re is not locally concentrated into a single structure, or a small number of structures, which means that Re is present in all cell types. In previous studies, it was demonstrated that the cell surface is not the main accumulation site of Re in the brown macroalgae *Pelvetia fastigiata* [9]. As a result, it would be expected that Re enters into the cell and remains in the cytoplasmic or a cell compartment. Moreover, Xiong *et al.* [15] made a macroalgae cell gel by chemically modifying brown macroalgae with sulfuric acid, obtaining a gel of the macroalgae alginate and fucoidan matrix. The resulting gel had a high Re affinity, and it was stated that amino acids were taking part in Re absorption, as it was observed in the IR (i.e. infrared) spectra that the intensity of the peaks corresponding to amino –NH_2_ groups decreased after adsorption. Moreover, this fact was supported by removal of the amino acids of the gel (i.e. previously boiling the brown algae) which showed no adsorption of Re. Thus, this could mean that Re is not found in the cell wall in macroalgae, but interacts with cell membrane proteins or other molecules that contain –NH_2_ groups in the cell, while not interacting with cytoplasmic proteins (figure 8). As in this study, no disruption of the membranes was carried out, it cannot be assumed that membrane bound proteins were simultaneously extracted. Moreover, the method for protein detection used does not detect free amino acids, peptides (i.e. glutathione, metallothioneins and phytochelatins) and proteins smaller than 3 kDa. Thus, it cannot be stated absolutely that Re is not protein bound, because we cannot be sure to have isolated all the proteins, but it can be stated that it is not related to cytoplasmic proteins larger than 3 kDa or, if it is, the Re binding of the protein is sufficiently weak that the analytical protocol for protein isolation is capable of breaking any Re protein associated bond.

### 4.2. Comparison of perrhenate compounds (HReO_4_, NaReO_4_, KReO_4_ and NH_4_ReO_4_) uptake by cultured *Fucus vesiculosus* tips

A sorption study of Re onto organic polymers was undertaken by Kim *et al.* [17], who concluded that negatively charged perrhenate ions interacted with protonated amine groups in chitosan. The authors explain the sorption by a combination of a Langmuir–Freundlich-type mechanism and the electric diffuse double layer model. Our experiments show that all perrhenate salts have the same linear trendline (figure 3*a*) which strongly differs from perrhenate obtained from HReO_4_ (figure 3*b*). This unexpected result highlights the importance of the chemical species of Re compound used for doping, which we further discuss below.

Perrhenate salts (NaReO_4_, KReO_4_ and NH_4_ReO_4_) are highly soluble in water with solubilities around 1.1 g ml^−1^. It has been observed that cations are used as a symport for perrhenate uptake in animal cells [20]. Our results seem to show that H^+^ is the best counter ion for perrhenate uptake; therefore, a greater uptake is observed when HReO_4_ is used. Moreover, H^+^ could be increasing the conversion of –NH_2_ groups of the macroalgae to $-{\mathrm{NH}}_{3}^{+}$, thus allowing perrhenate to bind. Therefore, more polymers of glucosamine and amino groups in *F. vesiculosus* [15,18] could be positively charged allowing more perrhenate binding, as it has been observed that perrhenate interacts strongly with polymers of glucosamine [17] and amino groups [15]. Although the difference of such discrepancy cannot be resolved here, uptake of ${\mathrm{ReO}}_{4}^{-}$ is observed no matter what form of perrhenate compound is used. The mechanisms that control Re entry into the cells of macroalgae have not been identified. There are many reports studying cation metal transporters, [38–40], but little is known about anion transporters (pumps) of macroalgae. Phosphate, chloride, sulfate, nitrate and molybdate transporters are all anion transporters reported in cells. Macroalgae could take up Re as perrhenate instead of other substrates of these transporters. Other trace metals in seawater exist, rather than as the free metal ion, as oxoanions (e.g. perrhenate, chromate, vanadate, molybdate, arsenate). The existing active transport pumps (e.g. sulfate, nitrate, phosphate) could be taking up such metal oxoanions, or there could be metal-specific pumps [41]. It has been observed that arsenate and phosphate have a common mechanism of uptake in bacteria and yeast [42], but not in phytoplankton [43] and brown macroalgae [19], although high concentrations of phosphate inhibit the uptake of arsenate. Nitrate could be also competing with perrhenate; however, this has been observed only for the mineral sodalite, and not in living organisms [22].

The seasonal Re(VII) salt uptake variation of cultures (table 6) suggest that perrhenate uptake is biologically influenced. Riget *et al.* [44] observed that zinc obtained maximum concentrations in macroalgae in March and a minimum in September, and it was similarly observed, albeit less clearly, with lead and copper. Macroalgae growth is the most likely cause for seasonal variations in metal uptake [44,45]. Although our studies seem to support this theory, a monthly perrhenate uptake research should be done in order to confirm it more strongly and decipher if it is simply a dilution effect or if perrhenate has a real metabolic role in the macroalgae. Here, we did not perform any seasonal experiments using HReO_4_.

Our study also shows that when non-fertile thallus tips start dying they do not accumulate more Re and start to degrade, thus Re is released to the media (table 6 and figure 4). Therefore, less accumulation of Re in those cultured macroalgae tips that started dying is expected. This happened in the macroalgae tips cultured with 2000 and 7450 ng g^−1^ of HReO_4_ in the seawater. In addition, it is worth emphasizing that the more time the dying tips are left in the water, the more Re is released in the seawater by macroalgae (i.e. the less accumulation of Re). Thus, this explains the results obtained in figure 4, where non-fertile thallus tips grown with a concentration of 2000 ng g^−1^ of HReO_4_ accumulate less Re than those cultured with 7450 ng g^−1^, because the first sets were cultured for 15 more days than the tips grown with 7450 ng g^−1^ of HReO_4_.

Therefore, a good linear correlation fit between HReO_4_ doped in seawater and Re taken up by *F. vesiculosus* is observed up to 75 ng g^−1^ Re in seawater, but with higher concentrations (i.e. 1000, 2000 and 7450 ng g^−1^), there is no linear correlation (figures 4 and 5) owing to the probable metabolic inactivation of the tips. This indicates that the limit of uptake by the tips occurs when the tips are grown in a media of between 75 and 1000 ng g^−1^ of Re.

Phytoaccumulation (or phytoextraction) of metals by plants and algae is widely known [46], and refers to the concentration of metals from the environment into plant tissues. Plants absorb substances through the root, and then they transport and store these substances into the stems or leaves. There are two types of phytoextraction species: accumulator species and hyperaccumulator species. The main difference between those two types is stated in Rascio & Navarri-Izzo [47]. Hyperaccumulator species are able to extract higher concentrations of metals and have a faster root-to-shoot transport system compared with non-hyperaccumulator species without showing phytotoxic effects. However, from the data obtained in this study, it cannot be stated that *F. vesiculosus* is a hyperaccumulator species, because the thallus tips grown with the highest concentrations of ${\mathrm{ReO}}_{4}^{-}$ started to decrease in growth and die; although they were at concentrations not typical of any environmental setting.

### 4.3. An understanding of Re uptake: active or passive

Figures 6 and 7 show that Re uptake is not by simple diffusion, as it is observed that only living *F. vesiculosus* tips concentrate Re. Re levels in tips with high Re media concentration (7.45 ng g^−1^) do not decrease when subsequently placed in media with lower Re concentrations: this suggests that the adsorption is not driven by simple equilibria. If Re was taken up by simple diffusion, then we would expect the same uptake of Re after boiling, freezing or drying the tips, as the membranes are not affected, and a direct correlation between the concentration of Re in the solution and in the macroalgae tips would be expected. Although Re could be taken up through passive mediated transport (facilitated diffusion), because after metabolically inactivating the macroalgae tips the transport proteins of the membranes are expected to be denatured (as happens when tips are boiled), thus no uptake is observed. However, this seems unlikely, owing to the high Re uptake observed in living *F. vesiculosus* tips relative to the Re concentration in seawater. In addition, our results show that the uptake mechanism is syn-life, therefore Re is bioabsorbed. It can also be concluded that Re is not taken up by simple diffusion, at least for the perrhenate compounds used here. Finally, figure 6 shows that the uptake mechanism of the macroalgae is unidirectional, not a simple partition, as we observe that once living *F. vesiculosus* has accumulated Re, it does not release it back to the media.

### 4.4. Implications of bioaccumulation of Re

Our results show little to no Re accumulation by metabolically inactivated *F. vesiculosus*, thus, if this is the case of macroalgae preserved in sediments as organic matter, using Re as a palaeoredox may not strictly apply. However, we do suggest that once *F. vesiculosus* has died we may see release back to the water column as the macroalgae breaks down. Thus, anoxia may be how the Re is stabilized, through prevention of macroalgae degradation.

Despite *F. vesiculosus* being a non-hyperaccumulator macroalgae, it is seen that until a limit, *F. vesiculosus* can accumulate up to 50 000 ng g^−1^ when HReO_4_ was present in the media, recovering the metal from the media. Thus, *F. vesiculosus* could be used as a source of phytomining of Re. Although differences in Re uptake are associated with the form of the perrhenate compounds, all ${\mathrm{ReO}}_{4}^{-}$ compounds used here permit the uptake of Re by *F. vesiculosus*. Moreover, as Re is also a Tc analogue [17], *F. vesiculosus* could be used for bioremediation of contaminated waters with Tc residues, as it has been found in ocean waters near to the Fukushima nuclear accident [48]. Tc is a radioactive metal, mainly artificially produced within nuclear reactors as a fission product of uranium and plutonium.

## 5. Conclusion

The observation that macroalgae concentrates Re, an element with no known biological use, raises interesting questions. This study documents the first detailed examination of the relative proportions of Re in the structures of the macroalgae. The following conclusions are drawn from this study.

1. Re is not solely concentrated into a single macroalgae structure, all the cells possess Re. There is a distribution of Re that increases from the holdfast to the tips. Non-reproductive thallus tips exhibit the most Re accumulation, even more than reproductive thallus tips. As the only difference between them is the reproductive structures (receptacles), we can say that Re is not bound in the reproductive structures.
2. Our data show that Re is bioadsorbed by *F. vesiculosus,* rather than bioaccumulated, and does not follow a simple diffusion uptake mechanism. The uptake is unidirectional, not a simple partition; however, the data show conclusively that *F. vesiculosus* takes up and stores Re.
3. Re recovery is observed from seawater enriched with ${\mathrm{ReO}}_{4}^{-}$, opening the possibility of using *F. vesiculosus* as a source of phytomining.
4. A difference in the uptake of Re between pherrenate salts and HReO_4_ is observed; however, the cause has yet to be established.
5. The seasonal differences in Re uptake associated with pherrenate salts are a function of *F. vesiculosus* growth.
6. There is a limit on the uptake of Re in the cultured macroalgae between 75 and 1000 ng g^−1^ of HReO_4_ in the seawater media, and beyond that a deleterious effect is observed.
7. Re is not accumulated in the cytoplasmic proteins or chloroplasts.

## Data accessibility

All the data that support the results in the article are included in tables 1–5 of this article.
